# Novel Lineage of Methicillin-Resistant *Staphylococcus aureus*, Hong Kong

**DOI:** 10.3201/eid1512.090378

**Published:** 2009-12

**Authors:** Luca Guardabassi, Margie O’Donoghue, Arshnee Moodley, Jeff Ho, Maureen Boost

**Affiliations:** University of Copenhagen, Frederiksberg C, Denmark (L. Guardabassi, A. Moodley); The Hong Kong Polytechnic University, Hung Hom, Kowloon, Hong Kong Special Administrative Region, People’s Republic of China (M. O’Donoghue, J. Ho, M. Boost)

**Keywords:** antimicrobial resistance, staphylococci, zoonoses, swine, Asia, MRSA, Staphylococcus aureus, Hong Kong, dispatch

## Abstract

To determine whether *spa* type of methicillin-resistant *Staphylococcus aureus* in pigs belonged to sequence type (ST) 398, we analyzed nasal swabs from pig carcasses at Hong Kong markets in 2008. ST9 belonging to *spa* type t899 was found for 16/100 samples, which indicates that a distinct lineage has emerged in pigs.

Methicillin-resistant *Staphylococcus aureus* (MRSA) has long been recognized as an important hospital pathogen and in recent years has emerged in the community. Increasing numbers of reports have concerned MRSA in animals. Multilocus sequence typing (MLST) has shown widespread dissemination of sequence type (ST) 398 among pigs in the Netherlands (*1*). This MRSA lineage has subsequently been reported in several other countries and animal species. Compelling microbiologic and epidemiologic evidence indicates that persons living or working on farms, especially pig farms, have an increased risk for colonization or infection with ST398 (*2*). In the present study, MRSA isolates obtained from slaughtered pigs in Hong Kong were characterized genotypically and compared with ST398.

## The Study

Nasal swab specimens collected by using Transwabs (Medical Wire Ltd, Corsham, UK) were collected from 100 pig carcasses at 2 wet markets in Hong Kong on 5 separate days over a 7-week period in 2008. Cross-contamination was minimized by selecting carcasses with intact nasopharyngeal tracts and by instructing the butchers taking part in the project to avoid causing damage to the nares when cutting up the heads. The frontal section of each snout was cleaned with 75% alcohol before swabbing the nasal mucosa up to 8 cm into the nares. Nasal swabs were enriched in brain–heart infusion broth (Oxoid Ltd, Basingstoke, UK) with 7% NaCl at 37^o^C for 48 h and then injected into mannitol salt agar (Oxoid) supplemented with 6 µg/mL oxacillin, for 24 h. Presumptive *S. aureus* colonies were tested for heat-stable nuclease (DNase) and coagulase production, and isolates positive for both were confirmed to the species level by latex agglutination (Staphaurex Plus, Murex Diagnostics Ltd, Dartford, UK). Antimicrobial drug sensitivity testing was performed by using disk diffusion following Clinical and Laboratory Standards Institute recommendations (*3*). Methicillin resistance was confirmed by disk diffusion using cefoxitin (30 μg) and *mec*A PCR detection. All MRSA isolates were characterized by pulsed-field gel electrophoresis (PFGE) by using *Sma*I (*4*), staphylococcal chromosome cassette (SCC) *mec* typing (*5*), and *spa* typing (*6*) with Ridom StaphType 1.4.1 software (www.ridom.de/staphtype). Two isolates representative of distinct PFGE patterns and SCC*mec* types were analyzed by MLST (*7*), and the remaining isolates were characterized by single-locus (*aroE*) sequencing.

MRSA was isolated from 16 samples collected on 4 of the 5 sampling days. In contrast to ST398, which has the characteristic of being nontypeable by PFGE using *Sma*I (*1**,**2*), the 16 MRSA isolates were typeable. They displayed 6 PFGE patterns; 2 predominant types (A1 and B1) were associated with SCC*mec* types IV and V, respectively (Table 1). Both PFGE types were ST9 according to MLST analysis. The 4 remaining patterns were either closely related to A1 (A2, A3, and A4) or possibly related to B1 (B2) according to the criteria of Tenover et al. (*8*). All isolates belonged to *spa* type t899 and harbored the ST9-associated *aroE* allele 3, which differs from that in ST398 (allele 35) by multiple mutations. Porcine MRSA ST9 isolates were negative for Panton-Valentine leukocidin genes and resistant to a broader range of antimicrobial agents than that previously described for MRSA ST398 isolated from pigs in the Netherlands (*1*). Twelve isolates displayed a typical multiple resistance pattern, including resistance to chloramphenicol, ciprofloxacin, clindamycin, cotrimoxazole, erythromycin, gentamicin, and tetracyline. The remaining 4 isolates were additionally resistant to fusidic acid (Table 1). All isolates were negative for Panton-Valentine leukocidin and susceptible to vancomycin and linezolid.

**Table 1:** Characteristics of methicillin-resistant *Staphylococcus aureus* isolates from pig carcasses in Hong Kong, 2008*

Isolate	Sampling date	Resistance pattern	*spa* type	SCC*mec* type	PFGE pattern†	*aroE* allele	MLST type
K1	Feb 22	3	t899	IVb	A1	3	ST9
G29	Mar 11	4	t899	IVb	A2	3	ST9‡
55	Mar 27	1	t899	IVb	A1	3	ST9‡
56		1	t899	V	B1	3	ST9
57		1	t899	V	B1	3	ST9‡
61		1	t899	V	B1	3	ST9‡
54		1	t899	V	B2	3	ST9‡
B40	Apr 15	1	t899	IVb	A1	3	ST9‡
B46		1	t899	IVb	A1	3	ST9‡
B50		1	t899	IVb	A1	3	ST9‡
B51		1	t899	IVb	A1	3	ST9‡
B52		1	t899	IVb	A1	3	ST9‡
B22		1	t899	V	B1	3	ST9‡
B39		1	t899	IVb	A3	3	ST9‡
B37		2	t899	IVb	A2	3	ST9‡
B36		2	t899	IVb	A4	3	ST9‡

*SCC, staphylococcal chromosome cassette; PFGE, pulsed-field gel electrophoresis; MLST, multilocus sequence typing. Resistance patterns: 1, oxacillin, penicillin, tetracycline, chloramphenicol, gentamicin, clindamycin, erythromycin, ciprofloxacin, cotrimoxazole; 2, oxacillin, penicillin, tetracycline, chloramphenicol, clindamycin , erythromycin, ciprofloxacin, fusidic acid; 3, oxacillin, penicillin, tetracycline, chloramphenicol, gentamicin, clindamycin, erythromycin, ciprofloxacin, cotrimoxazole, fusidic acid, 4, oxacillin, penicillin, tetracycline, chloramphenicol, clindamycin, ciprofloxacin, cotrimoxazole, fusidic acid. †Closely or possibly related PFGE patterns according to the Tenover criteria (*8*) are designated by the same letter and different numbers. ‡ST9 was predicted on the basis of PFGE and *spa* and *aroE* typing.

A search of the scientific literature and the Internet for information about the frequency of *S. aureus* ST9 in humans and animals indicated that ST9 is a clone of porcine origin. In 2005, Armand-Lefevre et al. (*9*) reported that ST9 was the most prevalent ST of methicillin-susceptible *S. aureus* (MSSA) isolated from pig farmers and infected pigs in France but not from a control group of persons without occupational contact with pigs. In 2007, an erythromycin-resistant MSSA ST9 clone belonging to *spa* type 337 was found to be endemic on a farm in Denmark (*10*). A clinical ST9 isolate of porcine origin carrying the multidrug resistance gene *cfr*, associated with linezolid resistance, has recently been described (*11*). Six ST9 sequences have been submitted to the MLST database (http://www.mlst.net), including 5 for MSSA isolates from bloodstream infections in the United Kingdom and 1 for a MRSA isolate from the nose of a pig in China (ID2357). Single cases of human infection with MRSA ST9 t899 have been reported in the Netherlands (*2*) and in Guangzhou, China (*12*). Eighteen *spa*-type t899 isolates have been submitted to the Ridom SpaServer (http://spaserver2.ridom.de): 10 from Germany, 7 from the Netherlands, and 1 from Belgium. All submissions were recorded as MRSA, but unfortunately, the origins of the isolates and the MLST types were not reported. Although MRSA t899 has been previously associated with ST398 isolates from pigs (*13*) and from participants at a conference on pig health (*14*), the repeat succession of this *spa* type is completely different from those of other ST398-related *spa* types and similar to those of *spa* types related to ST9 (Table 2). That the same *spa* type occurs in both ST9 and ST398 is surprising because the MLST allelic sequences of these 2 *S. aureus* lineages are unrelated (Table 2). However, the occurrence of the same *spa* types in distant lineages has been previously reported (*15*) and could have resulted from either convergent evolution or genetic recombination.

**Table 2:** Tandem repeat successions in *spa* types previously associated with *Staphylococcus aureus* ST9 and ST398*

*spa* type	Tandem repeat sequence	MLST allelic profile	ST
t899	07-16-23-----------------02-34	3-3-1-1-1-1-10	9
t337	07-16-23-23-02-12-23-02-34	3-3-1-1-1-1-10	9
t3198	07-16-16-23-23-02-02-12-23-02-34	3-3-1-1-1-1-10	9
t011	08-16-02---------25-34-24-25	3-35-19-2-20-26-39	398
t034	08-16-02-25-02-25-34-24-25	3-35-19-2-20-26-39	398
t0108	08-16-02---------25-----24-25	3-35-19-2-20-26-39	398
t567	08-------------02-25-----24-25	3-35-19-2-20-26-39	398
t571	08-16-02-25-02-25-34-----25	3-35-19-2-20-26-39	398

*MLST, multilocus sequence typing; ST, sequence type.

## Conclusions

Our study of MRSA colonization of commercial pigs in Asia provides evidence that methicillin resistance has emerged in a porcine *S. aureus* lineage other than ST398. It appears that ST9 has achieved methicillin resistance through multiple acquisitions of SCC*mec*, as indicated by the recovery of distinct PFGE and SCC*mec* types. A combination of the results of literature and database searches indicates that ST9 is associated with pig farming and, although it is found infrequently, this ST has been isolated from infected persons worldwide.

Several studies have previously investigated the prevalence of MRSA nasal carriage in pigs sampled immediately after slaughter (*2*) or at the farm of origin (*13*). In our study, samples were collected at wet markets, because it not possible to access pigs at the single slaughterhouse in Hong Kong or at the farm sites of origin because >90% of slaughter pigs are raised in mainland China and delivered by train directly to the slaughterhouse in Hong Kong. Notably, the previously reported human infection in China with MRSA-ST9 occurred in Guangzhou, the province closest to Hong Kong, where most of the pigs originate. The colonization rate determined in our study represents the level of contamination immediately prior to sale of pig meat to consumers. Although pig heads are rarely available in European and North American markets, because these parts of the animal are generally centrally processed, homemade soup using the pig’s nose is commonly consumed in Hong Kong; this gastronomic tradition may increase the risk for zoonotic transmission of MRSA. Further epidemiologic studies are needed to determine the rates of colonization and infection with MRSA and MSSA ST9 both in personnel exposed to pigs and in the community.
